# Supplementary material for: CsrA Regulates Swarming Motility and Carbohydrate and Amino Acid Metabolism in Vibrio alginolyticus
Source: Microorganisms. 2021 Nov 18;9(11):2383. doi: 10.3390/microorganisms9112383 (PMC8624728; doi:10.3390/microorganisms9112383)
Supplement: Supplementary file 1 [file microorganisms-09-02383-s001.zip › microorganisms-1442003-supplementary.pdf]

Table S1. Primers for construction in this study.

Purpose	Oligo name	5' to 3'
csrA Arg6His mutants construction	pSW7848_fwd	GTCTGATTCGTTACCAATTATGACAAC
	pSW7848_rev	GAATTCGATATCAAGCTTATCGATAC
	*csrA*R6H-up_fwd	ATAAGCTTGATATCGAATTCTGCACGCTCGCTTACCGG
	*csrA*R6H-up_rev	AAATTAGCATTCTTTGCTCCTTGAGTAATCTCTAAATTTTCTTGC
	*csrA*R6H-down_fwd	GGAGCAAAGAATGCTAATTTTGACTCACCG
	*csrA*R6H-down_rev	TAATTGGTAACGAATCAGACAGGATTCGAACCTCCGAC
	Del-check-pSW7848-F	TCACTGTCCCTTATTCGCACC
	Del-check-pSW7848-R	CTGCTTTTGAGCACTACCCG
	del-check-*csrA*-F	CACCCGAATGTGTGGATACAGC
	del-check-*csrA*-R	CTGGCTTAACCGTTGCTGATTTAAT
over expression of CsrA construction	over-*csrA*_fwd	GTCTGATTCGTTACCAATTATGACAAC
	over-*csrA*_rev	GAATTCGATATCAAGCTTATCGATAC
	over_pSCT32_fwd	ATAAGCTTGATATCGAATTCTGCACGCTCGCTTACCGG
	over_pSCT32_rev	AAATTAGCATTCTTTGCTCCTTGAGTAATCTCTAAATTTTCTTGC
	pSCT32-over-check-F	GGAGCAAAGAATGCTAATTTTGACTCACCG
	pSCT3-over-check-R	TAATTGGTAACGAATCAGACAGGATTCGAACCTCCGAC

Table S2. Primers for qPCR.

Oligo name	5' to 3'
q-*aceE*-F	TTTCCAGTCTTCTGCTGCGT
q-*aceE*-R	CATCAGTTTAGGGTGCGGGT
q-*acnB*-F	TTCAACGCGTAACTTCCCGA
q-*acnB*-R	CGTAGCGTCGATTTGCTTCG
q-*alaS*-F	CTTCCACAAGTTGGTTGGCG
q-*alaS*-R	TTAAGTCAGCTGGGAAGCCG
q-*ald*-F	TGGGTCGTTTGCCACTGTTA
q-*ald*-R	AATAGTGACGTCAGCGCGAA
q-*avtA*-F	GCGTGGCGTATTGATTGTCC
q-*avtA*-R	TTCGCGGTAAGCCTTTTCCA
q-*eno*-F	AAGACGCAGGTTACACAGCA
q-*eno*-R	CACGGTCAGAACGGCTCATA
q-*fbp*-F	CGTGATTTACGGCTCCTCCA
q-*fbp*-R	CACCCCTTGAGGGAAACGAA
q-*flaC2*-F	TTGAACGGCTCATTCGGTGA
q-*flaC2*-R	TGGGTCAACGCTCCAGTTTT
q-*flgC1*-F	CCCTTGCAAACGAAGAAGGC
q-*flgC1*-R	ATTGACCCATCTGCAGCGTA
q-*flgC2*-F	CAGAGAAGCGTTTTGAGCCG
q-*flgC2*-R	AGCAGACCTTGCTGCATACT
q-*fruA*-F	TGAACGGCCTGACTGACTTC
q-*fruA*-R	CTTTGTTTACCGGGCCTCCT
q-*gcvP*-F	CTTCACGCCGAACGTGATTC
q-*gcvP*-R	GCGTTCGCGATTTCCATACC
q-*glgA*-F	CGCGCAGGACTTAGTTGGTA
q-*glgA*-R	AGCTTTCTTCCCTTGCGACA
q-*cs*-F	GCGGATAAGAAAGCGACCCT
q-*cs*-R	ACCATTGGCTCCCAGCTTAC
q-*lafA*-F	GCACACTAACTACGCTTCGC
q-*lafA*-R	TGTTTGCGCTTCTAGACGGT
q-*pdhA*-F	CAATGTGCCGCCGATTTCTT
q-*pdhA*-R	GATGAGTGTTGCGCCTTTCC
q-*pfkA*-F	AATCGGTGGTGATGGTTCGT
q-*pfkA*-R	TCACGTAGACGGTCGATTGC
q-*proB*-F	ACGAGATGGCGAGTAAGCAG
q-proB-R	TCGCAACGGCATCATTTTCG
q-*proQ*-F	AAACCAGGTGCAGTACGTGT
q-*proQ*-R	GGTTTCGCTTTCGCCTTACC
q-*proV*-F	ACTTGCCGGCATCGATGTTA
q-*proV*-R	CAGGCTAACGCTCTCGCTAA
q-*putA*-F	CAGGCTGTAAACCGTCTGGT
q-*putA*-R	GTGAAACCTTTGTCGCGCAT
q-*sdaA*-F	AACGCGTCTATCTCTGGTGC
q-*sdaA*-R	ACCTGAACCTGACCTGCAAC
q-*sdaB*-F	GGCAAGTCGTCACTTCTCCA
q-*sdaB*-R	CAACTTCTCCCTGACACCCC
q-*sdaC*-F	AACCCTGAAGCGGACATCAC
q-*sdaC*-R	ACCGAATACTACGCCTGCTG
q-*serA*-F	AGAGGGACTCCACCCTTCTT

q-*serA*-R	CGGGAGCGGATACCAATGAA
q-*tdh*-F	AATCGGTGACCGCGTATCTG
q-*tdh*-R	AAGTGCTGTGTGTACTGCGT
q-*thrA*-F	CCGCAGGTAACGAGAAAGGT
q-*thrA*-R	TTCCACTAAACGAGGGTCGC
q-*ppc*-F	TGCGCATGTTAGGCCACTTA
q-*ppc*-R	GCTGTCCCTGTCTGCTTGAT
q-*pckA*-F	CGCCCGACAGAAGAAGAGTT
q-*pckA*-R	GATAAGTTGCATGCGCTCGG
q-*sdhA*-F	ATGCGTTGGGAAAACAGCAC
q-*sdhA*-R	AGACTTGTCCGCTAGATGCG
q-*sucA*-F	AGCATCCTACACCACGCAAA
q-*sucA*-R	AGTCTACAGAGTGCAACGCC
q-*sucC*-F	ACCAAACTGACGCTAACGGT
q-*sucC*-R	GGCGTTTCTTCCGCAACTTT
q-*fbaA*-F	CAGTGGGCTACTTGGGATGG
q-*fbaA*-R	CCATACGCGTGGATCGTAGT
q-*gltD*-F	GTATGACGCGCTGCCATTTT
q-*gltD*-R	TTGAGGTACGAACGCAGTCC
q-*gltB*-F	TTGTTGCCATTCTTGGTGCG
q-*gltB*-R	CAGCATGACTCGACCCTGTT
q-*ppsA*-F	TCAGAAGGTGAAACGGGCTACG
q-*ppsA*-R	TCAAGACGAGCAAGACCAAC
q-*pykA*-F	CACTACGCTGGGTCCTTCAA
q-*pykA*-R	TGGTACGGACTTTGTTCGCA
q-16S-F	CTGGAACTGAGACACGGTCC
q-16S-R	CTCGCACCCTCCGTATTACC
q-*recA*-F	TCTTCATCAACCAAATCCGTA
q-*recA*-R	GCTGCAATCTTATTCTTAACAACTT
q-*csrA*-F	AATTTTGACTCGCCGCGTAGG
q-*csrA*-R	TGAATGCGCATGTAGATTTCTTCACG

Table S3. Genes for q-PCR and mRNA stability experiments.

Gene_ID	Symbol	Description
BAU10_RS22550	*lafA*	lateral flagellin LafA
BAU10_RS02985	*flgC1*	flagellar basal-body rod protein FlgC
BAU10_RS16785	*flgC2*	flagellar basal-body rod protein C
BAU10_RS03035	*flaC2*	flagellin
BAU10_RS04120	*glgA*	glycogen synthase
BAU10_RS00575	*fbp*	Fructose-1,6-bisphosphatase
BAU10_RS14165	*pfkA*	6-phosphofructokinase
BAU10_RS12575	*fbaA*	fructose-bisphosphate aldolase
BAU10_RS12400	*eno*	phosphopyruvate hydratase
BAU10_RS19230	*fruA*	PTS fructose transporter subunit IIBC
**BAU10_RS12545**	***serA***	**D-3-phosphoglycerate dehydrogenase**
**BAU10_RS20470**	***pdhA***	**ABC transporter permease**
**BAU10_RS12140**	***aceE***	**pyruvate dehydrogenase (acetyl-transferring), homodimeric type**
**BAU10_RS12035**	***acnB***	**bifunctional aconitate hydratase 2/2-methylisocitrate dehydratase**
**BAU10_RS03265**	***CS***	**citrate (Si)-synthase**
**BAU10_RS13680**	***ppc***	**phosphoenolpyruvate carboxylase**
**BAU10_RS15770**	***pckA***	**phosphoenolpyruvate carboxykinase**
**BAU10_RS03280**	***sdhA***	**succinate dehydrogenase catalytic subunit**
**BAU10_RS03290**	***sucA***	**2-oxoglutarate dehydrogenase, E1 component**
**BAU10_RS03300**	***sucC***	**succinyl-CoA synthetase subunit beta**
**BAU10_RS17220**	***ppsA***	**phosphoenolpyruvate synthase**
BAU10_RS09350	*pykA*	pyruvate kinase II
BAU10_RS08610	*sdaC*	serine transporter
BAU10_RS08615	*sdaA*	L-serine dehydratase 1
BAU10_RS16735	*sdaB*	L-serine ammonia-lyase
BAU10_RS19180	*gcvP*	glycine dehydrogenase (aminomethyl-transferring)
**BAU10_RS01440**	***thrA***	**bifunctional aspartate kinase /homoserine dehydrogenase I**
**BAU10_RS22370**	***tdh***	**L-threonine 3-dehydrogenase**
**BAU10_RS06505**	***proV***	**ABC superfamily (glycine/betaine/ proline transport protein)**
BAU10_RS07350	*proQ*	RNA chaperone ProQ
BAU10_RS02410	*proB*	gamma-glutamyl kinase
**BAU10_RS23405**	***putA***	**bifunctional proline dehydrogenase/L-glutamate gamma-semialdehyde dehydrogenase**
**BAU10_RS04925**	***ald***	**alanine dehydrogenase**
BAU10_RS12335	*alaS*	alanine--tRNA ligase
BAU10_RS15215	*avtA*	valine--pyruvate transaminase
**BAU10_RS01380**	***gltD***	**glutamate synthase**
**BAU10_RS01385**	***gltB***	**glutamate synthase**

Note: Bold letters (depicted in black) indicate the genes used for mRNA stability experiments.
